# Simulated tears of antero-posterior rotator cuff force-couple and reduced glenoid concavity decrease anterior glenohumeral stability: a robot-assisted biomechanical analysis of a load and shift sequence

**DOI:** 10.1007/s00402-026-06383-4

**Published:** 2026-06-18

**Authors:** Mats Jonas Karlsfeld, Jens Wermers, Stefanie Tänzler, Julia Sußiek, Elmar Herbst, J. Christoph Katthagen

**Affiliations:** 1 Department of Trauma, Hand and Reconstructive Surgery, University Hospital Münster, Münster, Germany; 2 Münster University of Applied Sciences, Münster, Germany

**Keywords:** Shoulder instability, Rotator cuff, Glenoid depth, Biomechanical analysis, Robot-assisted, Human cadaveric specimen

## Abstract

**Backround:**

The glenoid concavity and the compression applied by the rotator cuff (RC) are essential for glenohumeral stability (GHS). This study aimed to determine how different simulated rotator cuff tears (RCT) and the glenoid depth influence GHS.

**Methods:**

A Load and Shift sequence was performed with eight fresh-frozen cadaveric shoulders in a robotic-assisted setup. Differently configured static loading of the reinforced RC and deltoid muscle (DLT) simulated intact RC and anterior, superior, anterosuperior, posterosuperior, mass, and complete RC plus DLT tears. Anterior dislocation forces and their changes were determined as indicators of stability. The glenoid depth and Bony shoulder stability ratio (BSSR) were defined as indicators of concavity. To assess GHS, the maximal force (*F*_*avg*_), the maximal force increase (*dF*_*avg*_), and their mean deviations (*ΔF*_*max*_, *ΔdF*_*max*_) to the intact configuration during the sequence were evaluated.

**Results:**

Simulated tears of the subscapularis tendon (SCP) (*ΔF*_*max*_ = 7.90 N, *ΔdF*_*max*_ = 1.54 N/mm) and simulated tears of the infraspinatus (ISP) + teres minor (TM) tendons (*ΔF*_*max*_ = 7.19 N, *ΔdF*_*max*_ = 1.48 N/mm) resulted in greater differences to the intact shoulder than simulated tears of the supraspinatus tendon (*ΔF*_*max*_ = 3.82 N, *ΔdF*_*max*_ = 1.16 N/mm). High correlations were observed between maximal force concerning glenoid depth (*r* = 0.81) and BSSR (*r* = 0.79).

**Conclusion:**

Simulated tears of the SCP or ISP + TM significantly affect the anterior GHS in this model. These findings highlight the importance of careful evaluation regarding the indication for surgical reconstruction in such configurations. Decreased glenoid concavity reduces the anterior GHS and should be considered in treatment algorithms for shoulder instability.

**Study design:**

Controlled Laboratory Study.

**Level of evidence:**

IV.

## Introduction

The ball-socket-joint configuration and the influence of various stabilizers of the glenohumeral joint lead to a system with a high range of motion but high rates of instabilities and dislocations [5, 23]. Static stabilizers are the labrum, the joint capsule, the glenohumeral ligaments, the bone congruency, the glenoid version, overall scapular geometry, and the negative glenohumeral pressure [5, 36]. The rotator cuff (RC), the scapulothoracic muscles, the neuromuscular control, and the proprioception are dynamic stabilizers [5]. The function of the RC and the deltoid muscle (DLT) as “force couples” centering the humeral head in the glenoid was described by Inman in 1944 and specified by Burkhart et al. [6, 17] The contribution of the “transverse force couple” comprising the subscapularis (SCP), infraspinatus (ISP), and teres minor (TM) muscles to the glenohumeral joint motion was demonstrated [39]. Through various biomechanical studies and computer simulations, the importance of the RC for glenohumeral stability (GHS) and motion has been proven [13, 14, 21, 26, 32, 33, 39]. Clinical studies underline the stabilizing function. In the presence of rotator cuff tear (RCT), patients with shoulder instability showed a significantly increased risk for re-dislocations [10]. On the other hand, shoulder dislocations can cause RCT - potentially a self-reinforcing system [44]. Due to the high recurrence rates reasoned by bone loss, the scientific focus was on glenoid and humeral bone defects, their characteristics, and their best possible treatment [7, 34]. The importance of glenoid concavity for GHS was recently brought into focus by Moroder et al. and confirmed in biomechanical studies [28–30, 35], 43– [42]. The concavity compression applied by the RC is the dynamic partner of the static concavity to ensure stability [5, 9, 25, 30]. Low glenoid depth may reduce GHS, which increases the force required for the RC to counterbalance this deficit and lead to higher rates of RCTs [27]. As an innovation to the previous studies, an advanced, robot-assisted cadaver model with attached soft tissue was used to evaluate the findings of the biomechanical study by Wermers et al. regarding the influence of glenoid concavity and the computer simulation by Steenbrink regarding the influence of various RCTs on GHS in a more realistic model [38, 42]. The purpose of this study was to determine which configurations of RCTs are particularly salient to the GHS and, as such, should be addressed by surgery to avert consequences such as re-dislocation or development of post-traumatic osteoarthritis. The study evaluated the influence of glenoid concavity as the passive counterpart of the active stabilizer RC. The Two main hypotheses of our study were that a tear of the strongest, isolated tendon, the SCP, has a higher impact on anterior GHS than other single tendon RCT and that a distinct glenoid concavity increases the GHS.

## Methods

**Fig. 1:**
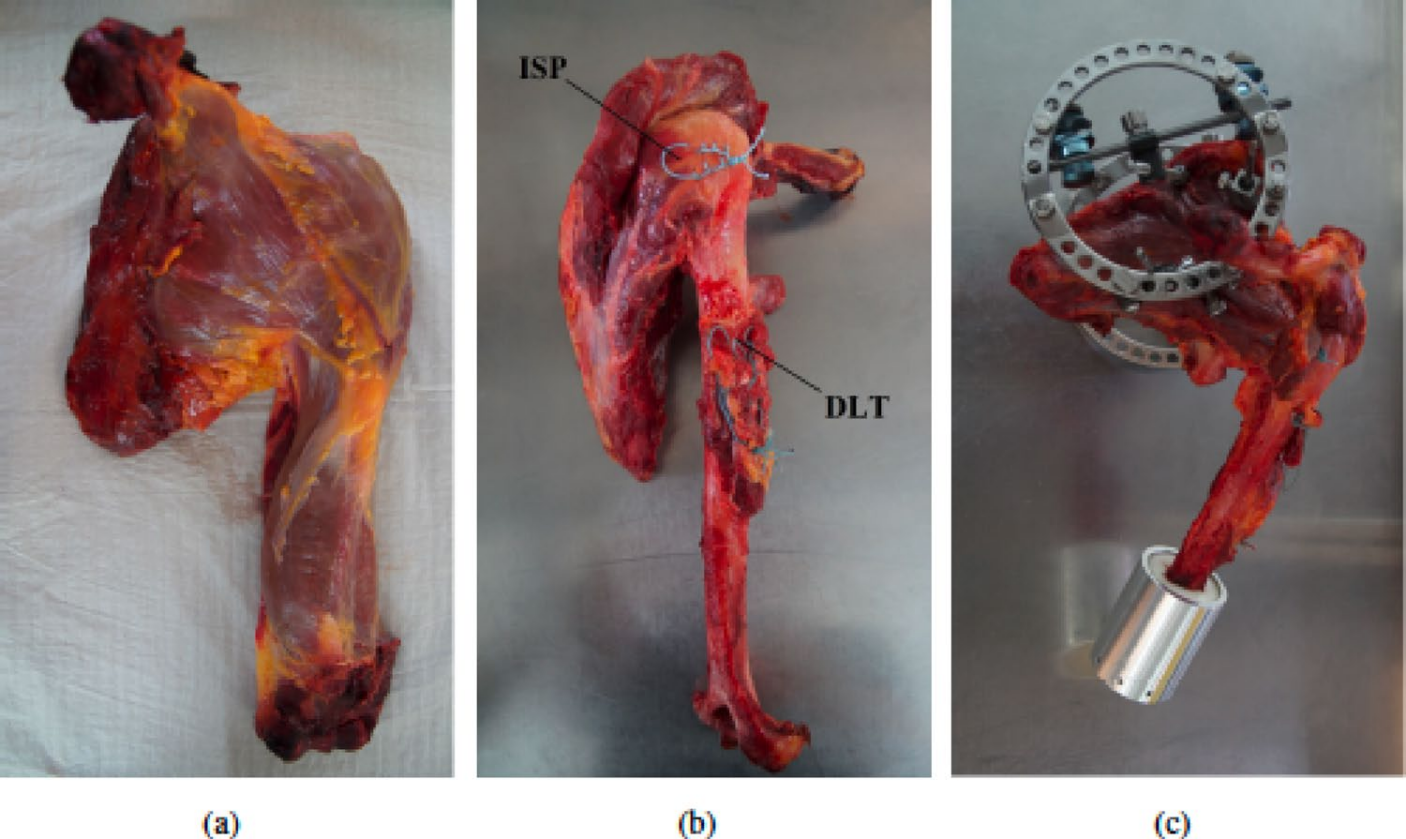
**a** removal of skin and fatty tissue; **b** preservation of the rotator cuff, DLT and biceps brachii muscle, reinforcement of ISP and DLT; **c** embedding of the distal humerus and fixation of the scapula via Schans pins with fixator rings

**Fig. 2:**
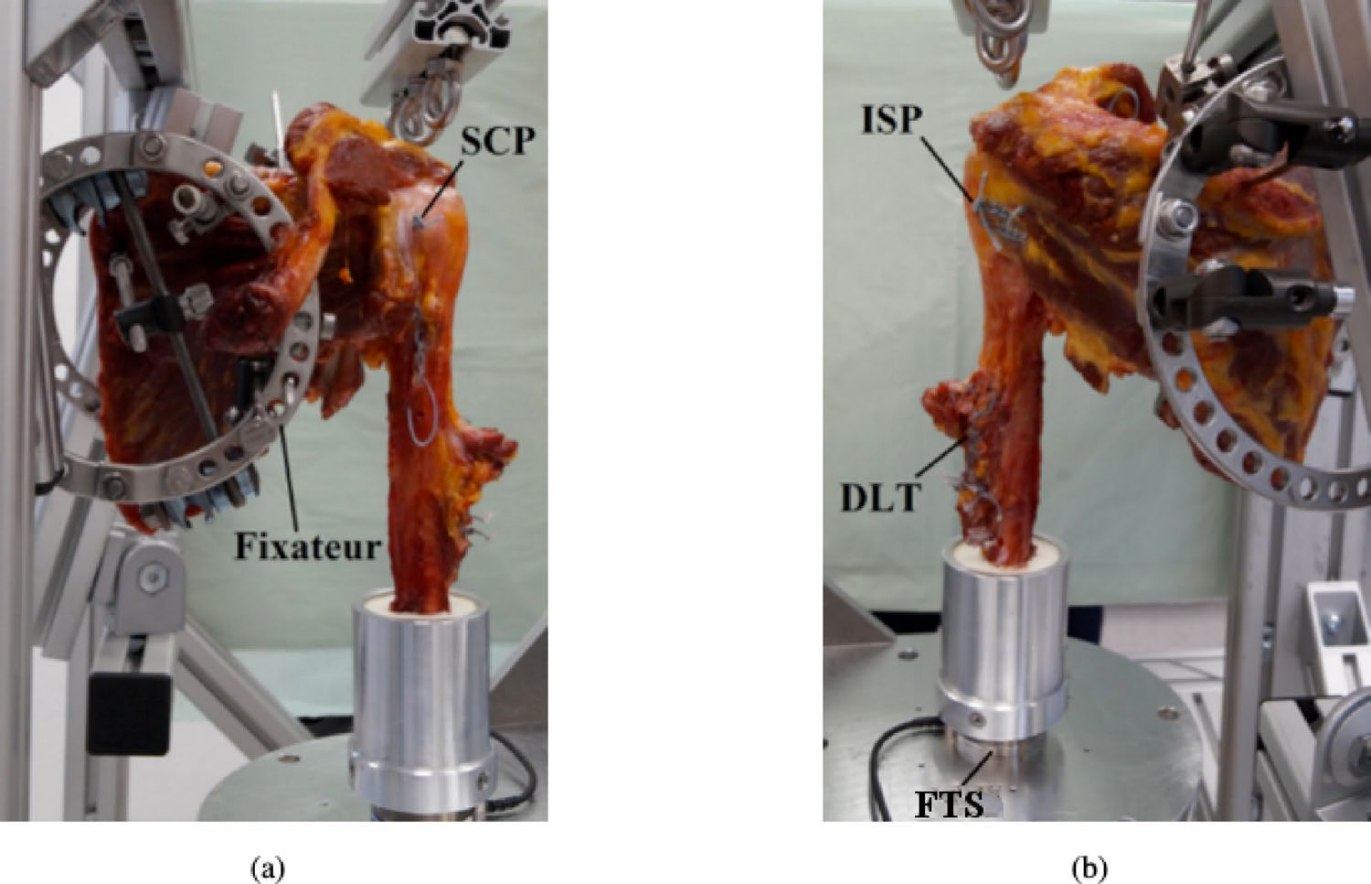
Fixation of the human specimen in the experimental setup and positioning of the force-torque sensor on the robot flange: **a** ventral view; **b** dorsal view

**Fig. 3:**
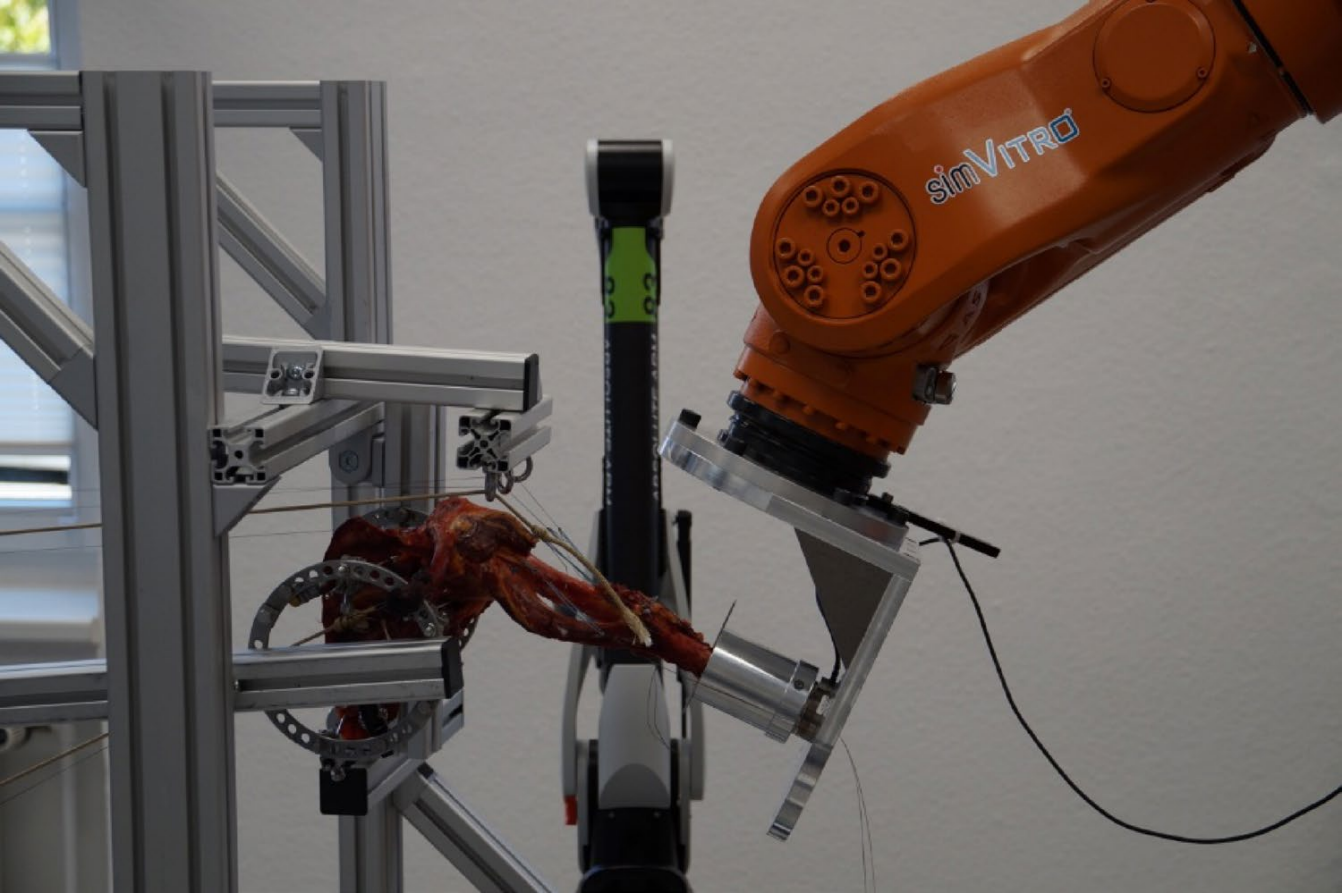
Final test setup for performing the Load&Shift sequence with the cadaver shoulder positioned in 60° abduction with weight-loaded parts of the DLT and the RC as well as the FTS inserted between the humerus and the robot arm

The study was approved by the local IRB (No. 2014-421-f-N, Germany, Münster). Seventeen fresh-frozen human shoulders obtained from body donors were initially considered for inclusion. No upper or lower age limit was applied. Specimens were excluded if they showed any of the following: glenohumeral osteoarthritis, osteophytes, or macroscopic or imaging-based evidence of damage to the RC, the DLT, or the long biceps tendon (LBT). Screening for these criteria was performed by macroscopic inspection and by morphological assessment of CT and MRI scans of each specimen. Based on this screening, nine specimens were excluded. The remaining eight shoulders (three right, five left; one female, seven male; mean age 82.25 ± 8.12 years, range 76–98 years) constituted the final cohort tested in the main experiments. After inclusion, each specimen was freed of skin and adipose tissue while preserving and dissecting the supraspinatus (SSP), SCP, ISP, TM, DLT, and LBT. Three drill holes of the spina scapulae, five drill holes of the proximal humerus, and one drill hole each on the inferior and superior angulus were set. After these steps, the CT and MRI scans were performed on each human specimen. The distal humerus was removed. To load the RC (SCP, SSP, ISP, TM) and the DLT tendons with weights, they were reinforced with a FiberWire Suture (US 5, Arthrex, Naples, USA) using the “Krakow stitch” technique. The dorsal capsular window was opened to exclude the phenomenon of the vacuum effect [1]. The remaining distal shaft was embedded in polyurethane casting resin (RenCast PU, Gößl + Pfaff GmbH, Karlskron/Brautlach, Germany), and the scapula was fixed via four to five Schanzscrews to Ilizarov Fixateur Rings (Figs. 1, 2, 3).

A profile rail construction was developed that allowed the static loading of muscles during the Load and Shift test performance. For the fixation of the specimen, the fixateur rings attached to the scapula were clamped between two customizable profile rails. The distal humerus was installed on the robotic flange along with a force-torque sensor (FTS).

The simulation of physiological muscle pulls was done through eyelets and pulleys installed on the profile rails. Threads of the reinforced tendons were connected by swivels with fishing lines. The loading of the muscles was performed with static weights in relation to their cross-section ratio according to the studies of Halder et al. and Veeger et al. [11, 40]. The ISP was loaded together with the TM with 22 N, the SSP with 8.7 N, the SCP with 20.4 N and the three parts of the DLT with 14.1 N each.

The movement and the measurement of the resulting forces were performed by an industrial robot (KR 60 − 3, KUKA, Augsburg, Germany), a robot-specific software for joint testing (simVITRO, Cleveland Clinic BioRobotics Lab, Ohio, USA) and the FTS (Mini45, ATI Industrial Automation, Apex, USA).

### Coordinate system

To ensure joint-specific movements of the robot, a three-dimensional joint coordinate system (JCS) was defined for each specimen separately based on CT scans and tactile measurements of reference drillings using a 3D measuring arm (Absolute Arm 8320-7, Hexagon Metrology). The anatomical landmarks were the inferior angle of the scapula (AI), the trigonum scapulae (TS), the center of the humerus (COR) as well as the medial (EM) and lateral epicondyles (EL) of the humerus. Based on the known three-dimensional relationship between reference drillings and anatomical landmarks from CT data, the position of tactilely unreachable anatomical landmarks, such as COR or the midpoint (MP), between the dissected EL and EM, was calculated. After the software had combined the measured JCS, the humerus could be moved by the robot and forces and torques could be measured in the resulting JCS.

The robot was programmed to simulate a clinical Load and Shift sequence. The tests were performed in glenohumeral abduction of 60° (corresponding to 90° humero-thoracic elevation) and neutral glenohumeral rotation in which the capsule and ligaments are loose, and glenohumeral stability is provided mainly by the articular surface, allowing the mechanical contribution of the articular geometry and the rotator cuff force couples to be isolated from passive soft-tissue restraint [19]. In the starting position, the humeral head was centered in the glenoid by the robot with a compression force of 10 N. Subsequently, the tendons were loaded with weights in eight different configurations, simulating different types of RCTs in the following order:

1. intact RC: SCP, SSP, ISP, TM and DLT loaded.
2. superior RCT: SCP, ISP, TM and DLT loaded; SSP unloaded.
3. posterior RCT: SCP, SSP and DLT loaded; ISP and TM unloaded.
4. anterior RCT: SSP, ISP, TM and DLT loaded; SCP unloaded.
5. anterosuperior RCT: ISP, TM and DLT loaded; SSP and SCP unloaded.
6. posterosuperior RCT: SCP, TM and DLT loaded; SSP and ISP unloaded.
7. total RCT: DLT loaded, SCP, SSP, ISP and TM unloaded.
8. unloaded: SCP, SSP, ISP and DLT unloaded.

The width of the glenoid was evaluated from CT measurements. From the starting position, the humeral head was moved anteriorly by 90% of half the glenoid width by the robot, then moved, passing the starting position, by 50% of half the glenoid width posteriorly. The movement was performed at a speed of 1 mm/s and maintained continuous contact between the glenoid and humerus, allowing the lateral movement of the robot to evaluate the depth and shape of the glenoid. The movement of the humeral head was position-controlled in the anterior-posterior direction and force-controlled in the superior-inferior and lateral-medial directions. Thus, the compression force resulting from the muscle loading was adopted and maintained during the test. The inferior force was controlled to zero to allow a change in position in this direction so that the humeral head could seek the path of least resistance and, if necessary, dislocate under the coracoid. Dislocation during the trial would represent a secondary endpoint. The anterior-posterior forces, as well as the lateral-medial and superior-inferior translation of the humeral head, were recorded.

The GHS was evaluated with respect to the maximal anterior force as well as the maximal force increase. The chosen parameters have the advantage that the different glenoid widths and shapes were considered by evaluating the entire interval up to a translation of 90% of half the glenoid width. The maximal force is defined as the maximal force occurring anteriorly during anterior translation. To define the maximal increase in force, the force was determined in an interval of one millimeter up to the maximal anterior translation and presented as force per millimeter. The largest gradient was evaluated as the maximal force increase.

The depth of the cartilaginous glenoid was calculated as the difference between the minimal and maximal lateral translation. Linear regression was used to examine the relationship between depth and GHS by plotting the glenoid depth with the corresponding maximal force or maximal force increase for each fully loaded shoulder on a common graph.

The CTs performed after the preparation of the specimens were used to determine the glenoid depth (*d*) and the glenoid radius (*r*) and thus to calculate the Bony shoulder stability ratio (BSSR) according to Moroder et al. [30].

$$\:BSSR=\:\frac{\sqrt{1-{\left(\frac{r-d}{r}\right)}^{2}}}{\frac{r-d}{r}}$$

The cartilaginous glenoid depth represents the functional articular interface including the cartilage layer, whereas the BSSR is bone-based and reflects the underlying osseous geometry derived from CT data. Both measures were included as complementary indicators of concavity to evaluate the relative contributions of osseous geometry and the cartilage-inclusive functional surface to anterior glenohumeral stability.

For each specimen, the maximal force and the maximal force increase were described as a function of the BSSR. The BSSR only considers the compression and dislocation forces, but not the external forces such as tensile forces of the muscles. Therefore, the external muscle forces were initially neglected in this regression and the correlation between BSSR and an unloaded shoulder was analyzed.

### Statistical analysis

To compare the force maximal and the maximal force increase of each simulated RCT with the intact RC, the t-test for dependent samples was performed with a significance level of α = 0.05. The most influential muscle was defined by the mean values of the maximal force and maximal increase in force (*F*_*avg*_, *dF*_*avg*_). For each simulated RCT, the mean deviation was formed as the difference of the mean values to the intact shoulder *(∆F*_*max*_, *∆dF*_*max*_), the standard deviation (*σF*,* σdF*), and the significance p-value was determined. The impact of glenoid depth and BSSR on glenohumeral stability is illustrated by the linear regression between these variables and the maximum force, as well as the maximum force increase. All statistical analyses were performed using GraphPad Prism (9.3.1- San Diego, USA).

## Results

The data for the maximal force and the maximal force increase was generated for *n* = 8 specimens with corresponding configurations of the RC and DLT.

The result of the maximal force for each simulated rupture is significantly different from the maximal force of the intact RC with *p* < 0, 001.

**Fig. 4:**
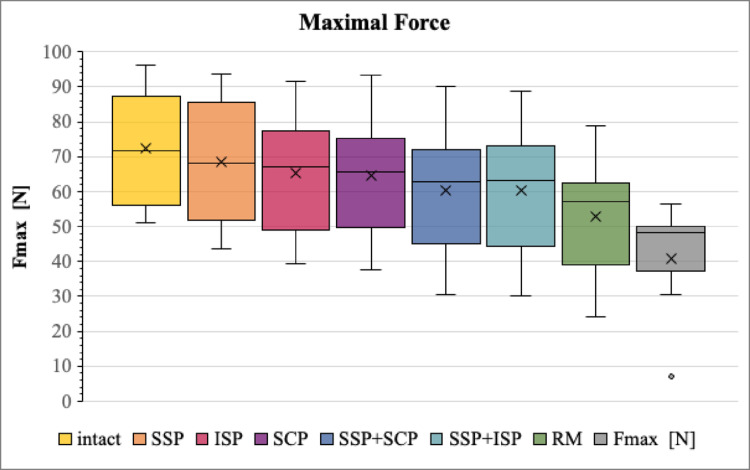
Effects of different configurations of the RC on the maximal force

The maximal force with intact RC + DLT is 72.37 ± 16.29 N. As the number of muscles involved in the simulated rupture increases, the occurring force during anterior translation decreases. In a total RCT, the maximal force is 52.77 ± 16.56 N. The mean difference (∆Fmax) from the intact RC + DLT configuration increases with the number of torn muscles. A simulated rupture of the SCP results in a mean difference of 7.9 N, a rupture of the SSP of 3.82 N, and a rupture of the ISP + TM of 7.19 N. The combined rupture of the SSP and SCP results in a smaller difference of 11.92 N than the combination of the SSP and ISP (∆Fmax = 12.05 N). A total RCT results in a difference of 19.6 N. The completely unloaded (RC + DLT) shoulder deviated by 33.89 N from the maximal force of the intact shoulder. The data on the maximal force can be taken from Fig. 4 as a boxplot and Table 1.

**Table 1:** Result of the dependent t-test for the maximal force: mean value and standard deviation of the maximal force for each individual condition; mean deviation as difference of the mean values of the individual ruptures and the intact shoulder and significance value p as probability of error

Configuration of RCT	Favg [*N*]	σF [*N*]	ΔFmax [*N*]	*p* (vs. intact) [1]
Intact	72,37	16,29	0	
SSP	68,55	17,33	3,82	≤ 0,001
ISP	65,19	17,18	7,19	≤ 0,001
SCP	64,48	17,21	7,90	≤ 0,001
SSP + SCP	60,46	18,13	11,92	≤ 0,001
SSP + ISP	60,32	18,35	12,05	≤ 0,001
RC	52,77	16,56	19,60	≤ 0,001
RC + DLT	40,94	15,67	33,89	≤ 0,001

### Maximal force increase

Analysis of the maximal force increase using the dependent t-test reveals a significant difference between the simulated ruptures and the intact condition with *p* ≤ 0, 05.

**Fig. 5:**
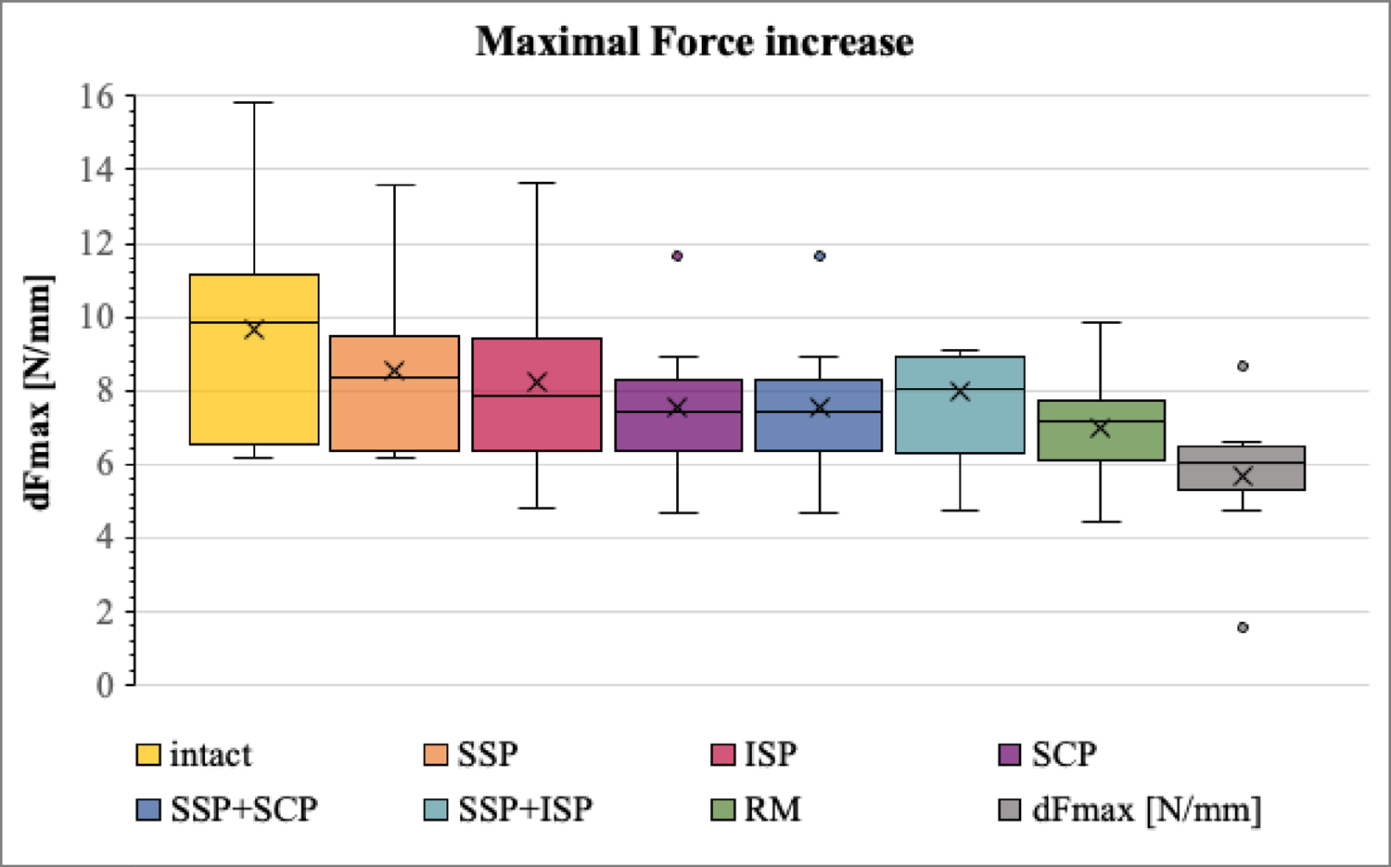
Effects of different configurations of the RC on the maximal force increase

The maximal force increase in the intact shoulder is 9.69 ± 3.15 N/mm. A single tendon RCT results in a maximal force increase of 8.53–8.15 N/mm and a total RCT of 6.98 ± 1.55 N/mm. The mean difference increases with the number of ruptures. A simulated rupture of a single RC tendon leads to a difference of 1.16–1.54 N/mm, a total RCT of 2.71 N/mm, and the completely unloaded shoulder differs from the intact shoulder by an average of 4.5 N/mm. When comparing the torn muscles of the RC, the SCP shows a greater difference of 1.54 N/mm than a rupture of the ISP (∆dFmax = 1.48 N/mm) and the SSP (∆dFmax = 1.16 N/mm). The combination of SSP and SCP results in a larger difference of 2.17 N/mm than a combined rupture of SSP and ISP (∆dFmax = 1.7 N). The maximal force increases are shown in Fig. 5 as a boxplot and Table 2.

**Table 2:** Result of the dependent t-test for the maximal force increase: mean value and standard deviation of the maximal force increase for each individual condition; mean deviation as the difference between the mean values of the individual ruptures and the intact shoulder and significance value p as the probability of error

Configuration of RCT	dFavg [*N*/mm]	σdF [*N*/mm]	ΔdFmax [*N*/mm]	*p* (vs. intact) [1]
Intact	9,69	3,15	0	
SSP	8,53	2,32	1,16	≤ 0,05
ISP	8,21	2,60	1,48	≤ 0,05
SCP	8,15	2,42	1,54	≤ 0,05
SSP + SCP	7,52	2,06	2,17	≤ 0,05
SSP + ISP	7,99	2,45	1,70	≤ 0,05
RC	6,98	1,55	2,71	≤ 0,05
RC + DLT	5,70	2,02	4,50	≤ 0,01

### Dependence of maximal force on the glenoid depth and BSSR

**Fig. 6:**
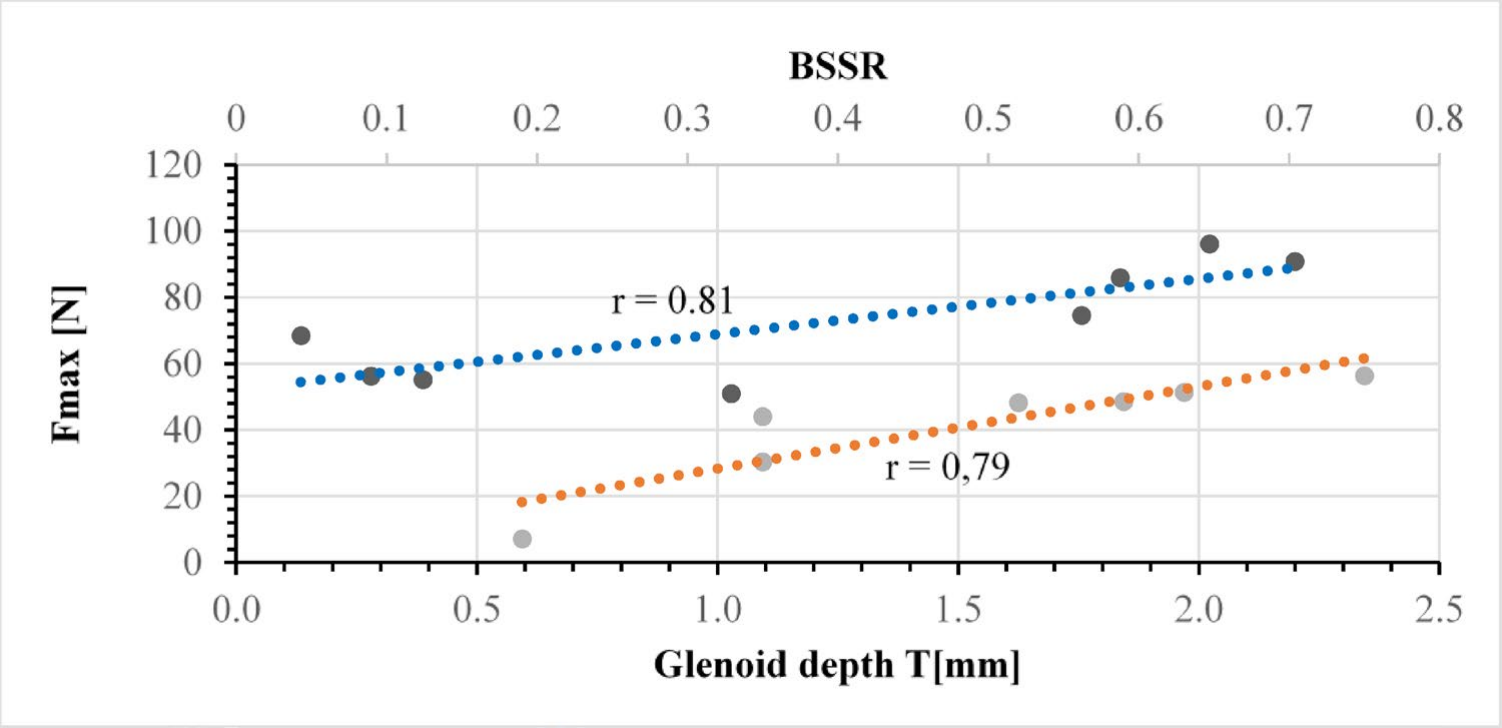
Maximal force as a function of BSSR and glenoid depth

The linear regression in Fig. 6 shows the influence of the maximal force on glenoid depth and BSSR. A correlation coefficient of *r* = 0,81 between maximal force and glenoid depth clarifies a high correlation. The dependence of the maximal force on BSSR shows a coefficient of *r* = 0,79.

### Dependence of maximal force increase on the glenoid depth and BSSR

**Fig. 7:**
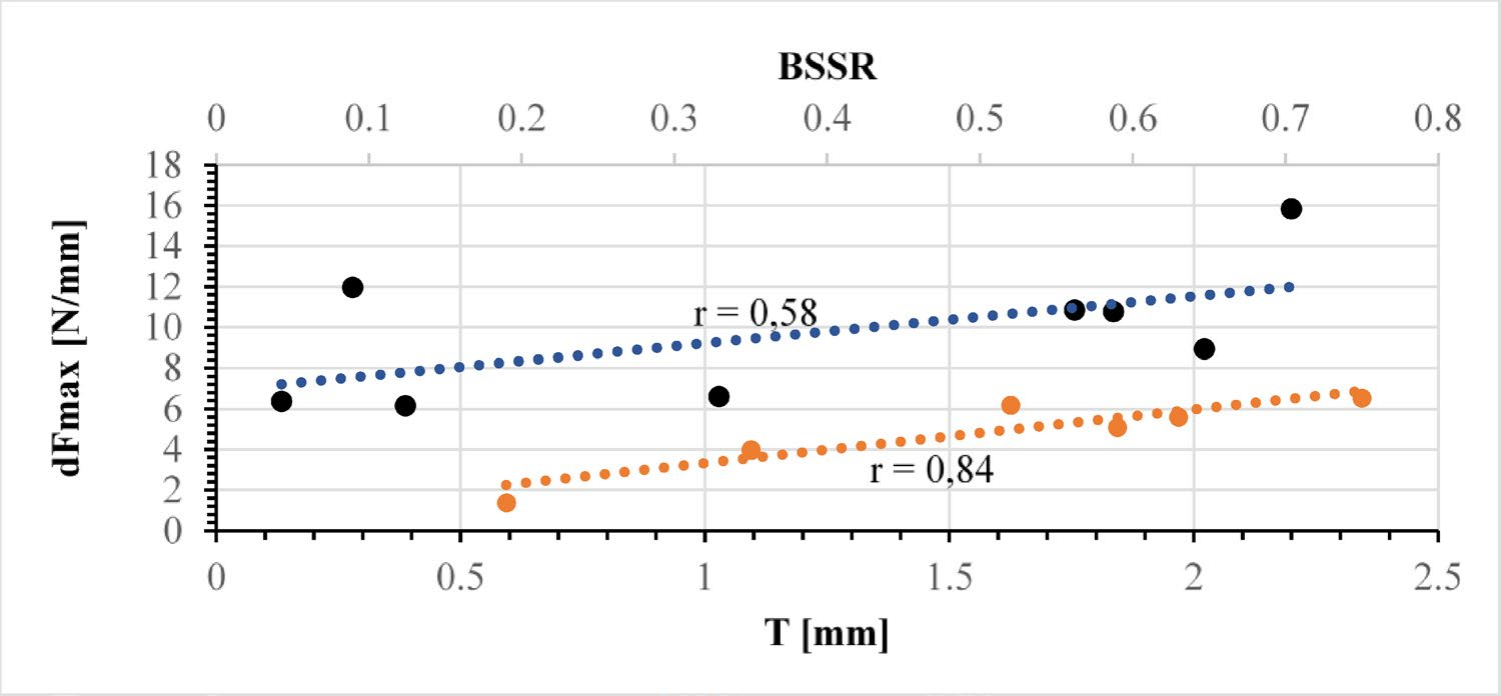
Maximal force increase as a function of BSSR and glenoid depth

The linear regression between maximal force increases on glenoid depth and BSSR is shown in Fig. 7. A correlation coefficient of *r* = 0,58 illustrates a distinct linear correlation on glenoid depth. The correlation between maximal force increase and BSSR presents a high correlation coefficient of *r* = 0,84.

## Discussion

This study showed that the maximal force and the maximal force increase of the dislocation force significantly depend on the condition of the RC. Ruptures of the SCP or the ISP + TM led to a higher reduction in GHS than a rupture of the SSP alone. Anterosuperior, posterosuperior or mass ruptures of the RC lead to the lowest GHS. The maximal force, as well as the maximal force increase, are linearly related to glenoid depth. Therefore, a more distinct glenoid depth increases GHS. The maximal force, as well as the maximal force increase, can be described as a function of the BSSR. Due to the linear relationship, the selected stability parameters can be estimated by measuring the radius and depth of the glenoid. The strength of these correlations supports the use of the BSSR as a relative indicator of glenohumeral stability; as no clinically validated thresholds are provided by the present data, glenoid concavity should be interpreted as a relative rather than absolute parameter in the context of instability.

A simulated tear of the SCP or ISP + TM results in a lower maximal force and a lower maximal force increase than a simulated SSP tear. Therefore, this study confirms the crucial stabilizing role of the SCP, which is due to the anatomical location and the strength of the muscle [9, 20]. Burkhart defined the role of the RC and the DLT as “force couples” for centering the humeral head in the coronal and the transverse plane, and therefore to ensure the pivot kinematic of the joint [6, 9, 17]. In 1996, Thompson et al. proved the theoretical idea and underlined the importance of the “transversal force couple” for centering in a biomechanical study [17, 39]. Larger RCTs involving the SSP and the anterior half of the ISP impact GHS due to superior and posterior translations during the mid-range of external rotation [18]. The result of our study supports the high relevance and clarifies that an imbalance of the “force couples” leads to a significant reduction in the anterior GHS.

Moroder et al. developed the BSSR and showed the importance of the glenoid depth and concavity for GHS [28–30]. This theoretical work was supported by the biomechanical study of Wermers et al., which showed that glenoid depth has a higher impact on GHS than the size of a bony defect [42]. Maalouly et al. showed in their study of glenohumeral parameters that a low glenoid depth is associated with higher rates of RCT’s [27]. The authors assumed that low glenoid depth reduces GHS, increasing the force required for the RC to counterbalance this deficit [27]. This study underlines this assumption without considering the glenoid shape and the humeral radius: a greater glenoid depth leads to higher GHS. It should be noted that the correlation coefficient is *r* = 0.89 for the maximal force and *r* = 0.92 for the maximal force increase of the linear regression is lower than the correlation of BSSR in the study by Wermers et al. [42]. The reason for the difference could be due to the additional influence of the adherent soft tissue and illustrates the need to consider other parameters for evaluating the GHS than just the osseous structures. Hu et al. underlined the high value of the glenoid concavity measures as stability ratio regarding functional outcomes and re-dislocation [15]. 

Souleiman et al. showed that the cartilage decisively shapes the concavity of the glenoid and therefore has a high influence on stability [37]. In contrast to these results, the present study shows that cartilage-inclusive glenoid depth, measured with the robot arm and therefore with intact cartilage, as a parameter of concavity correlates less well with glenohumeral stability than bone-based BSSR when soft tissue is attached; nevertheless, a correlation coefficient of 0.81 for maximal force confirms a high correlation between cartilage-inclusive glenoid depth and GHS. This could be reasoned by excentric and inhomogeneous age-related wearing or softening of the cartilage in the donor’s glenoids.

Limited biomechanical data suggests that less glenoid retroversion or anteversion of the glenoid may reduce the anterior forces required to decrease anterior glenohumeral stability [36]. Therefore, a Modified Bony Shoulder Stability Ratio that includes glenoid version was developed by Hu et al. [16] This study does not include glenoid version, but it is important to be aware of its potential on GHS, especially in extreme manifestations, and to include it in possible treatment algorithms. Further stabilizers of the glenohumeral joint — including the labrum, joint capsule, ligaments, scapular geometry, and those already mentioned — were not explicitly investigated in this study in order to focus on the contribution of the rotator cuff force couples and glenoid concavity.

### Clinical applicability

The study results suggest that simulated tears of the SCP and ISP/TM substantially affect force-couple stability and therefore warrant individual case-based assessment of the indication for surgical reconstruction. This is important for younger and older patients, especially those with pre-existing joint instability. The risk-benefit ratio should still be considered in patients over 70 years of age [8, 24]. Other factors for the success of an RC reconstruction, such as fatty infiltration and retraction of the ruptured tendons, bone quality and tear size must be considered [4]. Reverse shoulder arthroplasty is an alternative option for treating glenohumeral instability, especially in cases of existing osteoarthritis or non-reconstructable Soft-tissue [12]. In the case of rupture of SSP and ISP or SSP and SCP, the force balance should be analyzed for treatment indication [3, 6]. In case of imbalance with resulting unstable pivot kinematics, surgical intervention should be considered even in elderly patients. In treatment algorithms for shoulder instability, the glenoid concavity evaluated by CT or MRI in the form of the modified BSSR, BSSR or cartilaginous Shoulder Stability Ratio should be considered [30]. In borderline glenoid bone loss, where size-based criteria alone are equivocal, additional assessment of the remaining glenoid concavity may help refine the decision between non-operative management, soft-tissue stabilization, and bony reconstruction. Glenoid concavity may be crucial not only for the decision of bony reconstruction but also for the indication of RCT reconstruction [5, 7]. Patients with less glenoid concavity may benefit more from the stabilizing factor of RCT reconstruction. A distinct glenoid concavity may provide sufficient stability to make surgical reconstruction of the RCT unnecessary in elderly patients.

Although anterior shoulder instability classically presents in young, athletically active males, its incidence in patients aged ≥ 60 years is rising and is dominated by concomitant RCT in this older cohort [22, 31]. The mean donor age of 82.25 years therefore matches the demographic in which rotator-cuff-related instability is clinically most relevant; in younger patients, less pronounced cartilage wear likely preserves a higher functional concavity, but glenoid concavity remains a relevant factor to be considered across age groups [43]. 

The lack of muscle load did not fully replicate a true tendon tear: the muscle lost its function as an active stabilizer while its tendon remained as a passive stabilizer, so that the set-up corresponds to functional muscle unloading rather than a pathological tear [33]. For clarity, these conditions are accordingly referred to as ‘simulated tears’ throughout the manuscript. Static muscle loading was scaled to physiological cross-sectional area ratios to ensure reproducibility, but does not reproduce active muscle contraction, neuromuscular feedback, or muscular fatigue. The rigid fixation of the scapula precluded any scapulothoracic motion, although scapular kinematics and rotator cuff function are known to be interdependent [2]. Eliminating the negative intra-articular pressure by opening the dorsal capsule is expected to reduce the absolute anterior force values compared to the intact in vivo joint; because this condition was held constant across all test configurations, inter-condition differences and the relative ranking of force-couple contributions remain internally valid, whereas absolute force values should be interpreted accordingly. Human influence is a further potential source of error, as the investigators determined the muscle pulls individually for each specimen due to differences in clamping and anatomy. Given the limited sample size, the present analysis is regarded as exploratory, and the findings should be interpreted as indicative trends within the experimental model.

## Conclusion

Simulated tears of the SCP, as well as the ISP + TM, significantly affect GHS in this model. These findings highlight the importance of careful evaluation regarding the indication for surgical reconstruction in such configurations. Glenoid concavity has a strong influence on anterior GHS and should be considered in treatment algorithms for shoulder instability. Further studies could evaluate the influence of different RCTs and the glenoid depth on GHS in a dynamic experimental set-up and, therefore, various joint positions.

## Data Availability

No datasets were generated or analysed during the current study.
